# Methodological challenges when estimating the effects of season and seasonal exposures on birth outcomes

**DOI:** 10.1186/1471-2288-11-49

**Published:** 2011-04-18

**Authors:** Linn Beate Strand, Adrian G Barnett, Shilu Tong

**Affiliations:** 1 School of Public Health and Institute of Health and Biomedical Innovation, Queensland University of Technology, Australia

## Abstract

**Background:**

Many previous studies have found seasonal patterns in birth outcomes, but with little agreement about which season poses the highest risk. Some of the heterogeneity between studies may be explained by a previously unknown bias. The bias occurs in retrospective cohorts which include all births occurring within a fixed start and end date, which means shorter pregnancies are missed at the start of the study, and longer pregnancies are missed at the end. Our objective was to show the potential size of this bias and how to avoid it.

**Methods:**

To demonstrate the bias we simulated a retrospective birth cohort with no seasonal pattern in gestation and used a range of cohort end dates. As a real example, we used a cohort of 114,063 singleton births in Brisbane between 1 July 2005 and 30 June 2009 and examined the bias when estimating changes in gestation length associated with season (using month of conception) and a seasonal exposure (temperature). We used survival analyses with temperature as a time-dependent variable.

**Results:**

We found strong artificial seasonal patterns in gestation length by month of conception, which depended on the end date of the study. The bias was avoided when the day and month of the start date was just before the day and month of the end date (regardless of year), so that the longer gestations at the start of the study were balanced by the shorter gestations at the end. After removing the fixed cohort bias there was a noticeable change in the effect of temperature on gestation length. The adjusted hazard ratios were flatter at the extremes of temperature but steeper between 15 and 25°C.

**Conclusions:**

Studies using retrospective birth cohorts should account for the fixed cohort bias by removing selected births to get unbiased estimates of seasonal health effects.

## Background

Worldwide, it is estimated that 2.2% of all babies are stillborn [1] and 9.6% of all births are preterm (less than 37 completed weeks of gestation) [2]. Preterm babies are at greater risk of poor health and early death, require longer periods of hospitalisation after birth, and are more likely to develop disabilities [3-5].

Environmental and meteorological factors may be a cause of adverse birth outcomes [6]. Increases in air pollution [7] and temperature [8] have been associated with adverse birth outcomes. Air pollution and temperature usually have a strong seasonal pattern, meaning that one method of examining environmental factors is to explore seasonal patterns. Research has shown that the risk of preterm birth varies by season of birth [9,10] and season of conception [11]. Seasonal patterns of preterm birth differ from country to country, and peaks have been shown to occur at both hot and cold times of the year. For example, in a London cohort of almost 500,000 live singleton births, babies were more likely to be born preterm in winter than in spring (odds ratio = 1.10, 95% confidence interval (CI) 1.07-1.14), with the highest risk of preterm birth in November and December (probability of 0.76 per 1,000 fetuses at risk compared to 0.64 in April) [12]. However, in Greece preterm birth rates were highest in summer and spring [9], and in The Gambia preterm birth rates peaked in summer and autumn [13]. In Japan peaks of preterm births were reported in summer and winter [14]. The peak in winter was dominant among the northern latitudes and the peak in summer was dominant among the southern latitudes, suggesting that the seasonal pattern was dependent on factors differing between geographic locations (such as temperature) [15]. Different statistical methods may partly explain the differences in the seasonal patterns in the studies discussed above [16]. In particular, some studies assumed a sinusoidal seasonal exposure, whereas others looked at individual months or seasons.

Studies in developing countries have hypothesised that seasonal patterns in birth outcomes are due to seasonal changes in nutrition, infectious diseases, and seasonal work [13]. These factors are not as important in developed countries, but seasonal patterns in birth outcomes still occur, possibly because of seasonal changes in Vitamin D, air pollution or temperature [7,8,17,18]. As we show in this paper, seasonal patterns can also be due to biases in the selection of the study sample.

### The fixed cohort bias

**Figure 1:**
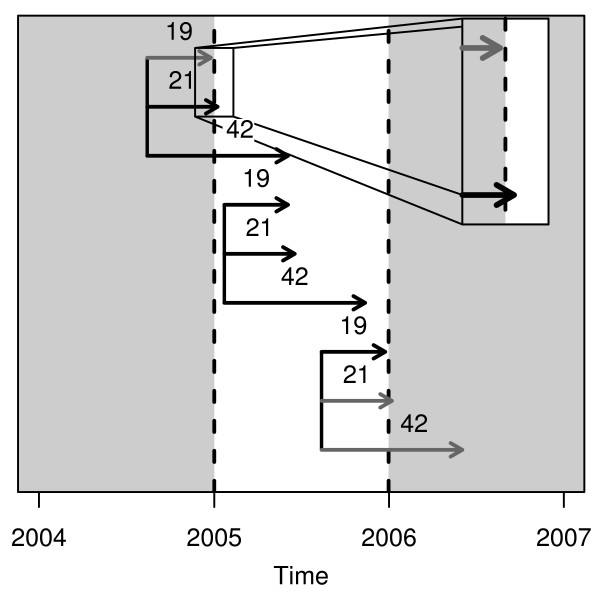
**How the population at-risk changes according to birth date using a birth cohort based on all births in 2005**. Black arrows show births included in the cohort and grey arrows show births that were missed. The number above each arrow shows the gestation length in weeks. The plot shows three groups of three births, each group represents one conception date.

For birth cohorts the population at-risk is constantly changing, as new pregnancies start and existing pregnancies end [19]. In retrospective birth cohort studies, when using a study period based on the date of birth (e.g., all births from 1 January 2005 to 31 December 2005), the population at-risk is different at the start and end of the cohort. The differences are shown in Figure 1 using three groups of three births, where each group has the same conception date. The shortest pregnancy registered in Brisbane statistical Division between 1 July 2005 and 30 June 2009 was 19 weeks and the longest was 43 weeks. For pregnancies conceived more than 19 weeks before the study start date, only those with a longer gestation will be included, and those with a shorter gestation will be missed because they gave birth before the start of the study. Similarly, for pregnancies conceived less than 43 weeks before the end of the cohort, mothers with longer gestations will give birth after the end of the study. Birth cohorts that prospectively follow women from conception to birth (or from their first antenatal visit to birth), do not experience this problem because the complete pregnancy history is known and no deliveries are missed.

The pattern shown in Figure 1 has the potential to bias seasonal patterns of gestation length by including only the longer pregnancies at the start of the study and only the shorter pregnancies at the end of the study. It also has the potential to bias studies of environmental exposures such as temperature. Just one example of how this could lead to biased estimates were if there was an unusually hot month in the first trimester of those women captured at the start of the study, which could mean that high temperatures were wrongly associated with longer gestations. We refer to this bias as the *fixed cohort bias*.

The arrows shown in Figure 1 call to mind to the well-known issue of left and right censoring in survival analysis [20], where either the start or end times are unknown. However, the fixed cohort bias is a different issue because it occurs when *both *the start and end times are unknown. These subjects will be missing from the at-risk population which will therefore be too small, and hence the consequences of the bias are similar to those caused by censoring.

The purpose of this paper is to demonstrate the potential effects of ignoring the fixed cohort bias, and to show how it can be avoided. We show the bias when estimating the effects of season, and the seasonal exposure of temperature.

## Methods

### The Brisbane cohort

We requested data on a cohort of all singleton births in the Brisbane Statistical District from 1 July 2005 to 30 June 2009 (n = 114,947) from the Queensland Health Perinatal Data Collection Unit. We only examined singleton births, so twins or triplets were excluded.

### Simulated cohorts

To investigate the fixed cohort bias we simulated a retrospective birth cohort with no seasonal pattern in gestation. The simulated cohort was based on the Brisbane cohort and had the same start and end dates (1 July 2005 to 30 June 2009). We randomly sampled gestation lengths from the Brisbane cohort (with replacement) using the original sample size (n = 114,947). We randomly simulated conception times from 43 weeks prior to the start of the study period (3 September 2004) until the end of the study period using a discrete Uniform distribution. Using these non-seasonal conception times and randomly sampled gestation lengths meant there was no seasonal pattern in gestation lengths or dates of births in the simulated cohorts, and that the distribution of gestation lengths was the same as the Brisbane cohort. Simulated births with a date of birth outside the cohort start and end dates were excluded, as these births would have been missed by a fixed cohort. Missing these births is the cause of the fixed cohort bias. The simulations were made using the R software (version 2.11.1).

### Statistical analyses

We ran a survival analysis of gestation length to analyse the effect of the fixed cohort bias on the seasonality of birth outcomes. We used a Cox proportional hazards model with a dependent variable of gestation length (in days) and an independent variable of conception month. A hazard ratio greater than one means an increased chance of giving birth, and hence shorter gestations. Whereas a hazard ratio less than one means a reduced chance of giving birth, and hence longer gestations.

We suspected that the effects of the fixed cohort bias would vary depending on the start and end dates of the study. To investigate this, we moved the end date of the study (using the simulated cohort) backwards in time (one month at a time) and repeated the survival analysis. We repeated this analysis for 100 simulated cohorts and calculated the average hazard ratios in each conception month.

We propose a method to avoid the fixed cohort bias by removing some pregnancies from the cohort. The adjusted cohort is created by limiting the included pregnancies to those with conception dates between:

1. 19 weeks before the cohort started, and

2. 43 weeks before the cohort ended.

We used 19 and 43 weeks as cut-offs since these were the longest and shortest gestation lengths observed in the Brisbane cohort. This ensures that the long pregnancies at the beginning of the cohort, and the short pregnancies at the end of the cohort, are excluded. We then repeated the survival analysis using the original Brisbane cohort and the adjusted cohort with month of conception as the single independent variable.

To investigate the effect of the fixed cohort bias on the effect of seasonal environmental exposures, we fitted a similar survival model but with the time-dependent variable of mean temperature in the last four weeks. We used polynomial splines for mean temperature [21]. The degrees of freedom of the spline control the degree of smoothness of the estimated temperature-gestation association. To allow for non-linear shapes, we used three degrees of freedom to describe the association between temperature and gestation length. We used the mean temperature in Brisbane (21°C) as the reference temperature where the hazard ratio was 1. We adjusted for mean humidity (in the last four weeks), baby sex, maternal age, Indigenous status, marital status, maternal smoking, number of previous pregnancies, and pregnancy complications (yes/no). We used both the original and adjusted Brisbane cohort. All models were fitted using the R software (version 2.11.1).

## Results

**Figure 2:**
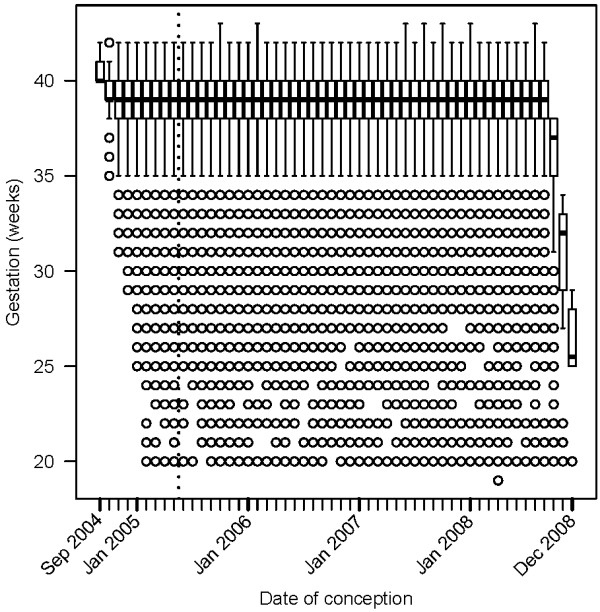
**Box plots of gestation (weeks) by month of conception for the Brisbane cohort**. Boxes show the inter-quartile range; outliers are circles. The dotted vertical line shows the start of the cohort based on birth dates (1 July 2005). The last birth was 30 June 2009 and the last gestation was in December 2008. Gestations for conceptions in September 2004 were all 40 weeks or longer, whereas those in December 2008 were all shorter than 30 weeks.

Table 1 describes the demographics of the original Brisbane cohort (n = 114,947). Figure 2 shows gestation lengths by month and year of conception. The births in the cohort conceived in September 2004 must have had a gestation of at least 40 weeks to be included, because any shorter pregnancies gave birth before the start of the study period (1 July 2005). The closer to the start of the study period, the more possible it became for women with shorter pregnancies to enter the cohort. In the middle of the cohort, gestation lengths were relatively stable, as all pregnancies were included and none were excluded because of their length. Towards the end of the cohort it became impossible for women conceiving during the study period but with longer pregnancies to be included, because they gave birth after the end of the study period. After removing the pregnancies conceived more than 19 weeks before the study start and less than 43 weeks before the study end, the adjusted cohort had 101,870 pregnancies (88.6% of the original sample).

**Table 1:** Demographics of the Brisbane cohort (births between 1 July 2005 and 30 June 2009)

	n	%
Total births	114,947	100.0
Gender		
Male	59,317	51.6
Female	55,630	48.4
Marital status		
Married/De facto	98,888	86.0
Never married	14,240	12.4
Separated/Divorced	1,788	1.6
Not stated	31	< 0.1
Indigenous status		
Indigenous	2,568	2.2
Non-indigenous	112,301	97.7
Not stated	78	0.1
Smoking		
Non-smoker	94,283	82.0
Smoker	19,845	17.3
Not stated	819	0.7
Pregnancy complications		
No complications	108,946	94.8
Pre-eclampsia	6,001	5.2
Previous pregnancies		
0	34,857	30.3
1-2	55,670	48.4
3-4	17,393	15.1
5+	7,027	6.1
Mother's age (years), mean (SD)	29.6 (5.8)	
Gestation length (weeks), mean (SD)	38.8 (2.3)	

SD = Standard deviation

### Simulation results

**Figure 3:**
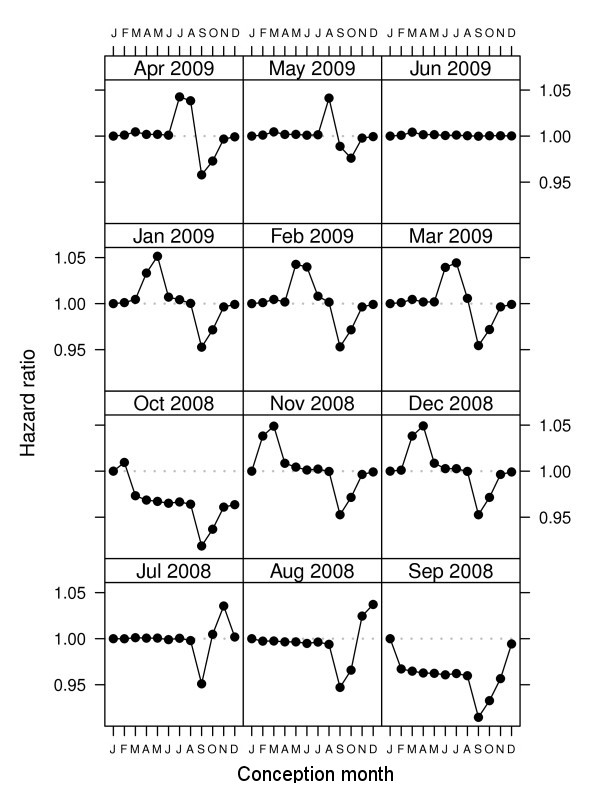
**Estimated seasonal pattern in gestation length by conception month for 12 end dates using 100 simulated cohorts (January is the reference month)**. Each panel shows the estimated seasonal pattern for a different end date. The start date was 1 July 2005. The data was simulated with no seasonal pattern.

Our simulated data had no seasonal pattern in gestation length. Figure 3 shows that an artificial seasonal pattern occurred for every end date except June 2009. The seasonal pattern varied according to the end date, so an end date of April 2009 meant that the shortest gestations were for conceptions in July (highest hazard ratio for giving birth), whereas an end date of January 2009 meant the shortest gestations were for conceptions in May (highest hazard ratio for giving birth). The bias was avoided when the day and month of the end date (31 June - ignoring year) were just before the day and month of the start date (1 July). This is because the shorter pregnancies at the end of the study period were balanced by the longer pregnancies at the start of the study.

### Brisbane results

**Figure 4:**
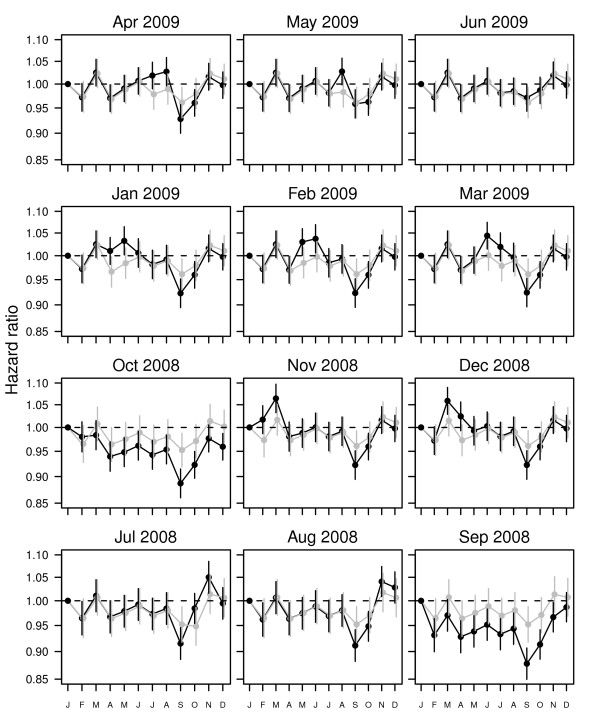
**Estimated seasonal pattern in gestation length by conception month for the Brisbane cohort**. Each panel shows the estimated seasonal pattern for a different end date. The black lines show the estimated seasonal pattern ignoring the fixed cohort bias. The grey lines show the estimated seasonal pattern after adjusting for the fixed cohort bias. Mean hazard ratios (dots) and 95% confidence intervals (vertical lines).

We further illustrate the fixed cohort bias using the Brisbane cohort in Figure 4. The estimated seasonal pattern in gestation length was strongly dependent on the end date of the study. For an end date of September 2008 the hazard ratios for many conception months were significantly lower than January (e.g., the hazard ratio for June relative to January was 0.95, 95% CI: 0.92, 0.98) meaning that gestations were longer for June conceptions. Conversely for an end date of March 2009 the hazard ratio for June relative to January was 1.04, 95% CI: 1.01, 1.07, meaning that gestations were shorter for June conceptions. After adjusting the cohort by removing births conceived more than 19 weeks before the cohort started, and less than 43 weeks before the cohort ended, the estimated seasonal pattern remained almost the same regardless of the study end date. The estimated seasonal pattern using the adjusted cohort was very close to that shown for an end date of June 2009, an end date which avoids the fixed cohort bias for this analysis because the end day and month are just before the start day and month (regardless of year).

### Temperature results

**Figure 5:**
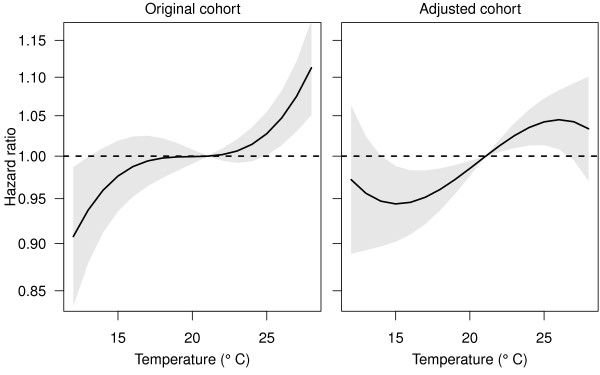
**The estimated effect of temperature on gestation length in the original Brisbane cohort (left) and the adjusted cohort (right)**. The solid line shows the mean hazard ratio and the grey area the 95 confidence limits. The dotted horizontal line at 1 represents no change in gestation length. The reference temperature is 21°C.

The effect of the fixed cohort bias in a study investigating temperature is shown in Figure 5. In the original cohort, longer gestations (reduced hazard ratios) occurred at temperatures from 12°C to 15°C, there was no change from the average gestation length between 15°C and 25°C, and average gestation lengths were shortened from 25°C and upwards. The estimated hazard ratios using the adjusted cohort were quite different. The hazard ratio of birth increased (gestation decreased) almost linearly from 15°C to 25°C. At very low and high temperatures the confidence intervals were wide, making it difficult to make any inferences.

## Discussions

Artificial seasonal patterns can occur in retrospective birth cohorts depending on the study's start and end dates (Figure 3). The cause is the great difference in pregnancy lengths between those births included at the start and those included at the end of the study. In our Brisbane cohort the median gestation length was 40 weeks at the start of the study, 39 weeks in the middle part of the study and as low as 25.5 weeks during the last conception month (Figure 2). The artificial seasonal patterns caused by the fixed cohort bias have a shape similar to an expected seasonal pattern, meaning that it could easily be wrongly attributed to a seasonal exposure (e.g., temperature). It is possible to remove the bias at the cost of a loss of sample size. We demonstrated how the bias was avoided by removing the pregnancies that were conceived earlier than 19 weeks prior to the start of the study period and later than 43 weeks before the end of the study period (Figure 4).

The biases are not caused by the well-known issues of censoring in survival data, which occurs when the start or end time of a subject are unknown. The bias demonstrated here is because both the start and end times are unknown. It is a bias caused by subjects who were completely (and unknowingly) missed from the cohort, not because of subjects who were partially observed.

The potential biases due to changes in the at-risk population when examining the effect of time-dependent exposures on pregnancy have been addressed by two recent studies [19,22]. These studies showed substantial biases due to ignoring seasonal patterns in pregnancies [19], and due to ignoring the week of gestation at study entry [22]. A recent study demonstrated the value of using time-dependent exposures as part of a Cox proportional hazards model when estimating the effects of air pollution on pregnancy, and concluded that this method was more effective than conventional approaches for estimating key exposure periods [23]. We also recommend the use of methods that adjust for the at-risk population and use time-dependent exposures to correctly estimate the effects of environmental exposures on pregnancy. However, we caution that when using these methods the fixed cohort bias needs to be considered. Our paper makes an important contribution to this developing area as it shows the potential size of the fixed cohort bias and a simple way to avoid it.

Our results show how the size of the fixed cohort bias can be substantial, causing great changes in the months that most effect gestation length (Figure 3, 4), and changing the estimated effect of temperature on gestation length (Figure 5). The results in Figure 3 showing the erroneous seasonal pattern were based on four years of data. To remove the seasonal pattern we excluded pregnancies with conception dates 19 weeks before the cohort started and 43 weeks before the cohort ended. These were around 13% of all pregnancies, and so represent a relatively large proportion of the sample, which explains the relatively large bias.

As shown in Figure 3, the fixed cohort bias is avoided when the day and month of the cohort's end date are a day before the day and month of the cohort's start date (regardless of year). A design with these 'matching' start and end dates is the most common for previous birth cohort studies [11,18,24,25]. However, the balancing of the bias will not occur for time-dependent exposures. For example, if there was an unusually hot month in the first trimester of those women captured at the start of the study, then high temperatures could be wrongly associated with longer gestations. The bias would only be avoided if there was an equally unusually hot month at end of the study period, when the women captured in the cohort have shorter gestations. We examined this potential bias in the original and adjusted Brisbane cohort and confirmed that the fixed cohort bias changed the effect estimates of temperature on gestation length (Figure 5). Ignoring the fixed cohort bias meant the biggest changes in the hazard ratios were at the extremes of temperature, whereas the adjusted estimate shows the biggest changes for more moderate temperatures. So if we ignored the fixed cohort bias we might wrongly advise that pregnant women avoid extreme temperatures (above 25°C), whereas the actual change in gestation length is for moderate temperatures (between 16 and 25°C). The fixed cohort bias can therefore not only bias the estimated effects of season (e.g., month of conception), but can also bias the estimated effects of seasonal exposures (e.g., air pollution and temperature).

A group of conceptions that were missed in this study were those occurring before 20 weeks (spontaneous abortions). These unfortunate cases are not added to the Brisbane birth registry. Rates of spontaneous abortions having reached at least five gestational weeks after last menstrual period vary from 11 to 16% [26-28]. Missing these cases has the potential to bias estimates of time-dependent exposures. Suppose an environmental exposure is strongly associated with spontaneous abortion, then a peak in this environmental exposure will cause an increase in spontaneous abortions and hence a decrease in the number of births that appear in the cohort. A study based on births after 19 weeks would miss the opportunity to detect the dangers of this exposure. Hence birth cohort studies should minimise their entry time, and clarify that the results only apply to pregnancies that have progressed to that time.

An ideal study design is one that prospectively follows women from close to conception, as this ensures that no pregnancies are missed (and hence the fixed cohort bias is not an issue). However, these designs are much more expensive that those relying on routinely collected birth data. A good alternative is to adjust cohorts using our method, and so avoid artificially created patterns and wrongly estimated effects from seasonal environmental exposures on birth outcomes.

Our results in Figure 5 are consistent with three previous studies that found that exposure to high temperatures in the week of birth increased the risk of preterm birth [8,9,29]. Maternal hyperthermia has been associated with abortions and stillbirths with lags of days to several weeks [30]. A study investigating hot tub and Jacuzzi use during the pregnancy and its effect on abortions found that pregnant women using hot tub or Jacuzzi during the pregnancy were twice as likely to have an abortion than women who did not (hazard ratio: 2.0, 95% confidence interval: 1.3, 3.1). Another study found that application of heat to the abdominal wall of women in labour increased uterine activity [31]. Dehydration during warmer temperatures may also be the cause of the shorter gestations, as insufficient fluids in the mother can decrease the amount of blood available to the fetus and induce uterine contractions [32]. Our results suggest that pregnant women should avoid exposure to high temperatures.

### Limitations

Although we controlled for Indigenous status we did not have data on other racial groups. As shown by Darrow et al [19] seasonal patterns in birth numbers by racial groups can cause seasonal patterns in birth outcomes. Therefore by only partially controlling for race the results in Figure 5 may be caused by a seasonal pattern in conception times in one or more racial groups. Also, we used data on ambient temperature and not the actual temperature experienced by the women which introduces a measurement error. For example, women with air conditioning would experience less exposure to high ambient temperatures.

We used a Cox proportional hazard model with a time-dependent exposure and therefore the results are presented as hazard ratios, and we were not able to give the estimated gestation length on the absolute scale of time (Figure 3). Using an accelerated failure time model or pooled logistic regression model [33] in place of the Cox model would mean the results could be given on a time scale.

## Conclusions

Correctly estimating the effects of environmental exposures on pregnancy is vital because an unhealthy start to life can mean an unhealthy adulthood. The adverse health effects of preterm birth and low birth weight include: socio-emotional and educational problems [3], reduced cognitive function [4], impaired vision and hearing [5] and restricted growth [34]. New research into the possible effects of the temperature on pregnancy is particularly important because of climate change. Future changes are predicted to include an increase in 'mega-heatwaves' such as those experienced in Europe in 2003 and 2010 [35]. If higher temperatures increase the risk of preterm birth as shown here (Figure 5), then we can expect a greater future public health burden due to preterm birth.

## Competing interests

The authors declare that they have no competing interests.

## Authors' contributions

LS and AGB discovered the bias and designed the method to avoid it. LS wrote the first draft of the manuscript. AGB ran the statistical analyses. ST supervised the study and contributed to the final draft. All authors contributed to study concept and design. All authors read and approved the final manuscript.

## Pre-publication history

The pre-publication history for this paper can be accessed here:

http://www.biomedcentral.com/1471-2288/11/49/prepub
